# Accelerated prime-and-trap vaccine regimen in mice using repRNA-based CSP malaria vaccine

**DOI:** 10.1038/s41541-023-00799-4

**Published:** 2024-01-10

**Authors:** Zachary MacMillen, Kiara Hatzakis, Adrian Simpson, Melanie J. Shears, Felicia Watson, Jesse H. Erasmus, Amit P. Khandhar, Brandon Wilder, Sean C. Murphy, Steven G. Reed, James W. Davie, Marion Avril

**Affiliations:** 1 MalarVx, Inc 1551 Eastlake Ave E, Suite 100, Seattle, WA 98102 USA; 2 HDT Bio, 1150 Eastlake Ave E, Suite 200A, Seattle, WA 98109 USA; 3 University of Washington, Department of Laboratory Medicine and Pathology, 750 Republican St., F870, Seattle, WA 98109 USA; 4 Vaccine & Gene Therapy Institute, Oregon Health & Science University, Building 1, Room 2220, 505 NW 185th Ave, Beaverton, OR 97006 USA

**Keywords:** Malaria, Parasitic infection, RNA vaccines

## Abstract

Malaria, caused by *Plasmodium* parasites, remains one of the most devastating infectious diseases worldwide, despite control efforts to lower morbidity and mortality. Both advanced candidate vaccines, RTS,S and R21, are subunit (SU) vaccines that target a single *Plasmodium falciparum* (Pf) pre-erythrocytic (PE) sporozoite (spz) surface protein known as circumsporozoite (CS). These vaccines induce humoral immunity but fail to elicit CD8 + T-cell responses sufficient for long-term protection. In contrast, whole-organism (WO) vaccines, such as Radiation Attenuated Sporozoites (RAS), achieved sterile protection but require a series of intravenous doses administered in multiple clinic visits. Moreover, these WO vaccines must be produced in mosquitos, a burdensome process that severely limits their availability. To reduce reliance on WO while maintaining protection via both antibodies and Trm responses, we have developed an accelerated vaccination regimen that combines two distinct agents in a prime-and-trap strategy. The priming dose is a single dose of self-replicating RNA encoding the full-length *P. yoelii* CS protein, delivered via an advanced cationic nanocarrier (LION^TM^). The trapping dose consists of one dose of WO RAS. Our vaccine induces a strong immune response when administered in an accelerated regimen, i.e., either 5-day or same-day immunization. Additionally, mice after same-day immunization showed a 2-day delay of blood patency with 90% sterile protection against a 3-week spz challenge. The same-day regimen also induced durable 70% sterile protection against a 2-month spz challenge. Our approach presents a clear path to late-stage preclinical and clinical testing of dose-sparing, same-day regimens that can confer sterilizing protection against malaria.

## Introduction

Despite control efforts that have reduced morbidity and mortality, malaria remains a major burden globally, with deaths and cases rising in recent years^1^. The SU vaccine RTS,S/AS01E, the only malaria vaccine approved by the World Health Organization (WHO), is composed of virus-like particles consisting of hepatitis B virus surface antigen (HBsAg) combined with a fragment of Pf CS protein. While approval of the RTS,S vaccine represents a significant step forward, it is unlikely to lead to eradication of malaria, given its modest efficacy, which declines further over years^2–6^. More recently, the SU R21-MatrixM vaccine, also approved for use in Ghana and using the same CS protein as RTS,S but with an increased ratio of CS to HBsAg, has also exhibited significant efficacy in a seasonal malaria setting. However, the utility of both RTS,S, and R21 vaccines is severely limited by the requirement for 3–4 doses plus seasonal boosters to achieve the WHO’s strategic goal of 75% protective efficacy against clinical malaria.

Development of SU vaccines against *Plasmodium* is challenging due to the parasite’s large genome and complex multi-host life cycle. Eliminating the parasite at the clinically-silent PE stage would prevent erythrocytic infection and thus halt both disease and transmission^7,8^. One of the primary candidate antigens for PE vaccine development is the CS protein, which is expressed by infectious spz and is required for parasite motility and hepatocyte invasion. Among other features, CS has an immunodominant central repeat region of four amino acids (NANP) that is the target of neutralizing antibodies, and a GPI-anchored C-terminal region containing a T-cell epitope^4,7,8^. Numerous vaccine platforms using CS have been evaluated as malaria interventions, but have not met the critical benchmarks^8–15^. Other strategies focus on delivery of WO vaccines (such as RAS immunization) that can elicit high antibody titers, central memory and effector memory CD8 + T cells (Tcm and Tem respectively), and resident (non-circulating) memory T cells (Trm)^16^. These Tem and Trm have been shown to mediate protection in WO-immunized mice by eliminating infected hepatocytes and conferring sterilizing immunity^17–19^. Protection has been achieved in both animal models and humans using repeated immunizations with WO RAS. Vaccination strategies employing WO spz (RAS, genetically-attenuated parasites (GAP), or chemoprophylaxis combined with administration of Pf spz (known as CPS)) have successfully protected malaria-naïve volunteers^20–24^. The protection appears to be mediated by a combination of liver-resident Trm and antibodies^25^. However, the WO RAS vaccination strategy was less effective in malaria-endemic regions, confirming the need for more robust approaches^26–29^.

The vaccination method known as “prime-and-trap” is intended to generate cellular immunity, by inducing liver Trm-mediated protection, as well as humoral immunity, with the help of CD4 + T cells and antibody production by B cells. Previous pre-clinical vaccination studies have assessed both homologous and heterologous prime-boost and prime-target approaches^9,30,31^ that have been shown to induce both specific, functional antibodies and high numbers of Trm at the time of infection, but none of these has achieved the strong safety profile of WO vaccination. Other “prime-and-trap” approaches combine a priming dose of a nucleic acid with a heterologous trapping dose of WO spz that naturally home to the liver^32,33^. The resulting liver Trm are positioned to respond effectively to liver-stage parasites, leading to sterile protection. Prime-and-trap vaccination using a gene-gun-delivered nucleic-acid prime and WO RAS trap in BALB/cJ mice completely protected against WT rodent malaria (*Py*) spz challenge^32^. Importantly, this approach required only one dose of WO spz^33^. However, the prime-and-trap vaccines developed to date have generally relied on DNA vaccination by gene gun^32^, and there is no gene gun delivery device currently approved for clinical use.

Lipid nanoparticles (LNPs) are highly effective means of delivering ribonucleic acid (RNA) vaccines. The use of antigen-encoding messenger RNA (mRNA) emerged as a promising strategy for vaccination during the COVID-19 pandemic: these vaccines express antigen upon delivery into tissue, stimulate the innate immune system, induce cellular immunity, and eliminate the need to support large-scale protein antigen production. When PfCS mRNA was combined with a lipid nanoparticle (LNP) and tested in mice, a three-dose regimen achieved up to 60% protection in a Pb(ANKA)-PfCS challenge model in BALB/c and C57Bl6^34^. Long-term protection was not observed, again highlighting the limitations of SU-only approaches. More recently, mRNAs encoding the PfCS and Pfs25 proteins were formulated with LNP and used to elicit functionally effective immune responses in mice to both antigens. This formulation protected against spz challenge and reduced Pf transmission to mosquitoes after multiple immunizations^35^. However, clinical trials with mRNA vaccines formulated with traditional LNPs^36–38^ have encountered challenges such as LNP/mRNA reactogenicity upon injection, biodistribution to multiple organs^39,40^, and instability during prolonged storage^41^.

Self-replicating or replicon RNA (repRNA) is a new and promising alternative to mRNA for use in vaccines. Once in cells, repRNA initiates biosynthesis of antigen-encoding mRNA, raising and prolonging antigen expression and enhancing humoral and cellular immune responses^42,43^. Based on recent studies^42,44,45^, repRNA systems formulated in an oil-in-water emulsion nanocarrier exhibit greater efficacy at lower doses than mRNA vaccine platforms. Moreover, repRNA elicits more robust immune responses after a single dose, offering an attractive approach for emerging infectious diseases such as dengue^45^, Zika^44^, Crimean-Congo hemorrhagic fever virus^46^, Mycobacterium tuberculosis^47^ and SARS-CoV-2/COVID-19^42^.

Here, we leverage this LION/repRNA technology to generate a two-dose, same-day prime-and-trap vaccine against malaria in mice. This was achieved by intramuscular (IM) priming with repRNA encoding full-length CS of *Plasmodium yoelii* (Py) (repRNA-PyCS) formulated in the LION nanoparticle carrier, followed by an IV injection of WO RAS as the trapping dose. This two-component, same-day regimen conferred sterile protection in mice and engaged both humoral and cellular arms of the immune system, bringing us closer to a single-visit, highly effective malaria vaccine.

## Results

### repRNA-CS vaccine formulation and prime-boost immunogenicity in BALB/cJ mice

**Fig. 1:**
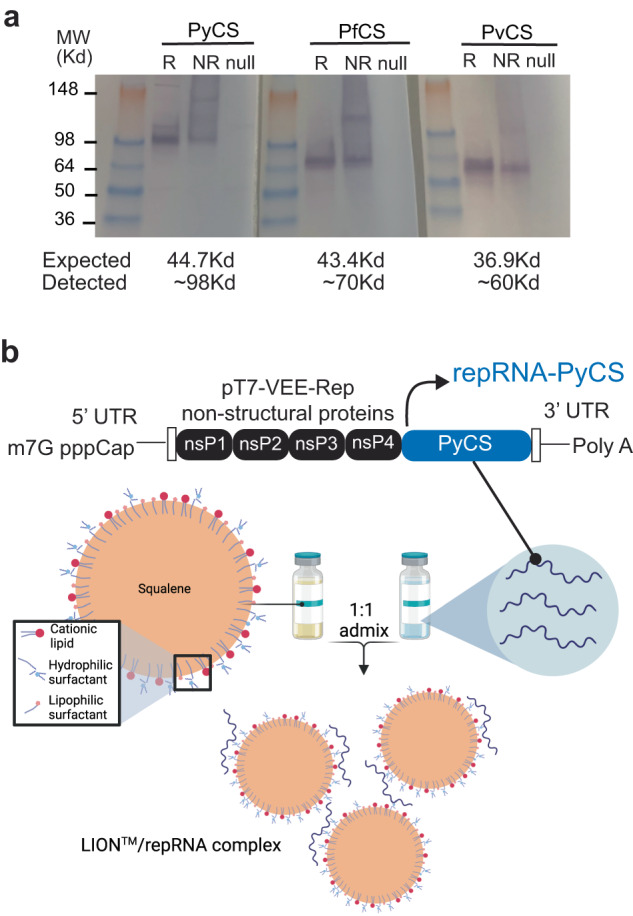
LION/repRNA-CS vaccine design. **a** After RNA transcription and capping, repRNA-PyCS or -PfCS or -PvCS was transfected into BHK cells. 24 to 48 h later, the lysate (R, reduced, NR, non-reduced) of transfected cells or null transfection used as control were analyzed by Western blot, using rabbit polyclonal antibodies for immunodetection. **b** Graphic representation of replicon repRNA-PyCS and LION formulation that was used for immunization after mixing.

Using the attenuated Venezuelan equine encephalitis (VEE) virus TC-83 strain^48^, we incorporated the coding sequence of the full-length CS protein from Py into the alphavirus expression vector to create a repRNA malaria vaccine. The coding sequences of the full-length CS protein from Pf and *Plasmodium vivax* (Pv) were incorporated into the same expression vector and were utilized as irrelevant RNA controls (Supplementary Fig. 1A). After RNA transcription and capping, integrity of repRNA-PyCS, -PvCS, and -PfCS was verified by denaturing gel electrophoresis (Supplementary Fig. 1B). The vectors were then transfected into mammalian cells for validation. Western-blot analysis showed expression of CS proteins at higher apparent molecular weights (MW) than expected (observed vs expected: PyCS ~99 Kd vs 44.7 Kd, PfCS ~70 Kd vs 43.4 Kd, PvCS ~60 Kd vs 36.9 Kd, respectively), probably due to glycosylation that impeded protein migration into the gel (Fig. 1a, Supplementary Fig. 1C).

The repRNA-CS vaccines were formulated with the LION emulsion (Fig. 1b)^42^. Unlike current mRNA vaccines, the LION/repRNA vaccine platform utilizes an admixture formulation of LION (Fig. 1b) that can be manufactured independently of the RNA component and combined with the repRNA 30 min prior to immunization. To determine the immunogenicity of homologous prime-boost LION/repRNA-CS vaccination with single or dual CS antigens, mice were immunized with an IM prime of 5 µg of either LION/repRNA-PyCS or LION/repRNA-PfCS (used as an irrelevant repRNA) as a single-antigen dose or a combination of both. Primed mice received a homologous boost 14 days later (Supplementary Fig. 2A). Final bleeds were collected three weeks post-boost and immune responses were analyzed by ELISA against the corresponding CS tandem-repeat region. Humoral immune responses against corresponding CS antigens as evaluated by ELISA exhibited little to no cross-reactivity against heterologous CS (Supplementary Fig. 2B). All mice seroconverted after immunization.

### Immunogenicity and efficacy of a two-dose prime-boost repRNA-PyCSP vaccine in BALB/cJ mice

**Fig. 2:**
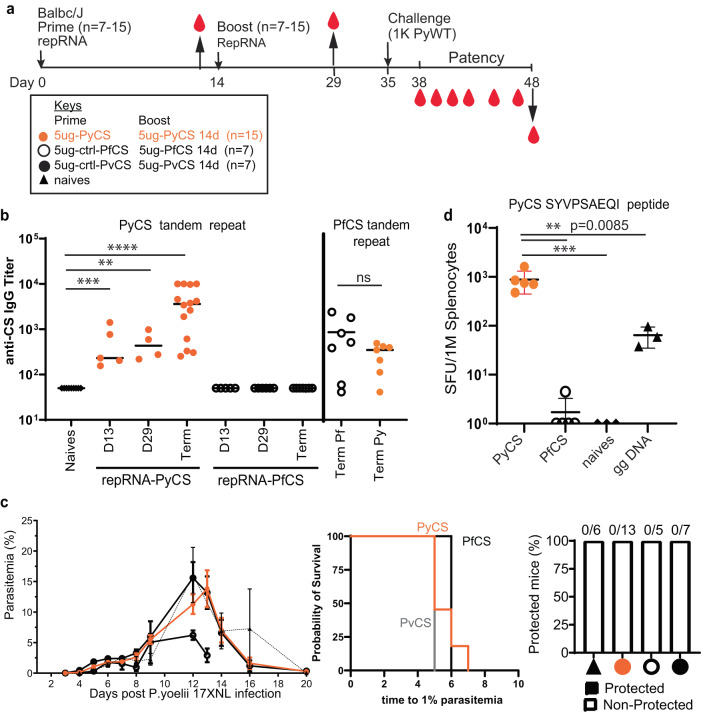
Immunogenicity and efficacy of a prime-boost vaccine. Vaccine designed with repRNA encoding either PyCS (orange circle), PfCS (opened circle) or PvCS (black circle) formulated with LION. **a** BALB/cJ mice immunization schedule. Mice were injected with 5ug of LION/repRNA-PyCS, -PfCS, or -PvCS in a 2-week interval prime-boost regimen. **b** Mouse sera were collected at post-prime (day 13), post-boost (day 29), and post-challenge (day 48, i.e., “term” as terminal bleed). PyCS and PfCS antibody responses were determined by PyCS or PfCS repeat region peptide titration ELISA, respectively. **c** Parasitemia, patency curves (>1% parasitemia) of mice post-challenge and protection post-challenge of the three prime-boost cohorts. Number of mice per cohort indicated above bar graph. Naïve cohort indicated with a triangle symbol and dash line. **d** IFNγ ELISPOT of CS-specific T cells (against the PyCS SYVPSAEQI epitope) four weeks after a single prime injection of LION/repRNA-PyCS. Cohorts receiving gene gun DNA encoding PyCS (ggDNA), LION/repRNA-PfCS, or no immunization were used as controls. The n value represents total number of mice tested per cohort, in two to three independent assays. Each data point represents an individual mouse and the bar represents the group mean. Asterisks represent significance as determined by the non-parametric two-tailed Mann–Whitney two-tailed test (*p = 0.05, **p = 0.01, ***p = 0.001, ****p < 0.0001).

To confirm antibody responses to homologous prime-boost LION/repRNA-PyCS vaccination in BALB/cJ, 15 mice were immunized with IM injections 14 days apart (Fig. 2a, Table 1). The LION/repRNA-PfCS and LION/repRNA-PvCS vaccines were used as separate irrelevant repRNA controls in cohorts of 7 mice each. Antibody responses were evaluated by ELISA following the prime (day 13) and boost (day 29) against peptides containing their corresponding CS tandem-repeat sequences (Fig. 2b). The anti-PyCS total IgG antibody levels in primed mice (D13, *p* = 0.001, Fig. 2b) were significantly higher than in naïve mice and were enhanced by a second dose of LION/repRNA-PyCS (day 29, *p* = 0.028, Fig. 2b) with slight cross-reactivity observed against the PfCS antigen (Fig. 2b).

**Table 1:** Summary of mouse immunization and challenge studies with *P.yoelii* wild-type parasites. Protection data were evaluated using Fisher’s exact test.

Immunization					Challenge				*p* value	Linked to
BALB/cJ mice	PRIME dose (LION/ repRNA)	Route	2nd dose (day given)	Route	IV dose (week given)	Blood-stage patency	Protected/ Challenged	% protection		
15	5μg PyCSP	IM	5 µg PyCSP (day 14)	IM	1000 WT SPZ (week 3)	4 days	0/13	0%		Fig. 2
7	5μg PfCSP	IM	5 µg PfCSP (day 14)	IM	1000 WT SPZ (week 3)	4 days	0/5	0%		
7	5μg PvCSP	IM	5 µg PvCSP (day 14)	IM	1000 WT SPZ (week 3)	4 days	0/7	0%		
13	5μg PyCSP	IM	25,000 RAS (day 14)	IV	1000 WT SPZ (week 3)	7 days	8/13	61%*	0.0186	Fig. 5
13	5μg PyCSP	IM	25,000 RAS (day 5)	IV	1000 WT SPZ (week 3)	7 days	10/13	77%*	0.0033	
11	1μg PyCSP	IM	25,000 RAS (day 14)	IV	1000 WT SPZ (week 3)	8 days	7/11	64%*	0.0174	
13	–	–	25,000 RAS	IV	1000 WT SPZ (week 3)	6 days	9/13	69%*	0.0085	
5	–	–	–	–	1000 WT SPZ (week 3)	5 days	0/5	0%		
					*Rechallenge					
					1000 WT SPZ (week 6)	none	6/6	100%		
					1000 WT SPZ (week 6)	none	6/6	100%		
					1000 WT SPZ (week 6)	none	6/6	100%		
					1000 WT SPZ (week 6)	none	6/6	100%		
10	5μg PyCSP	IM	25,000 RAS (day 5)	IV	10,000 WT SPZ (week 8)	7–8 days	5/10	50%	0.0325	Fig. 6
10	5μg PfCSP	IM	25,000 RAS (day 5)	IV	10,000 WT SPZ (week 8)	7–9 days	3/10	30%		
10	5μg PyCSP	IM	5 µg PyCSP (day 5)	IM	10,000 WT SPZ (week 8)	5–7 days	0/10	0%		
5	–	–	–	–	10,000 WT SPZ (week 8)	4–5 days	0/5	0%		
10	5μg PyCSP	IM	25,000 RAS (day 5)	IV	1000 WT SPZ (week 3)	6–7 days	8/10	80%	0.023	Fig. 7
9	5μg PyCSP	IM	25,000 RAS (day 0)	IV	1000 WT SPZ (week 3)	5 days	8/9	89%	0.014	
5	5μg PfCSP	IM	25,000 RAS (day 0)	IV	1000 WT SPZ (week 3)	6–7 days	2/5	40%		
7	–	–	–	–	1000 WT SPZ (week 3)	4 days	0/7	0%		
10	5μg PyCSP	IM	25,000 RAS (day 0)	IV	20,000 cryo SPZ (week 8)	7–8 days	7/10	70%	0.0098	
10	5μg PfCSP	IM	25,000 RAS (day 0)	IV	20,000 cryo SPZ (week 8)	7–8 days	3/10	30%		
7	–	–	–	–	20,000 cryo SPZ (week 8)	5 days	0/7	0%		
**C57Bl6**										
10	5μg PyCSP	IM	25,000 RAS (day 5)	IV	5000 WT SPZ (week 8)	4 days	0/5	0%		Supplementary Fig. 3
10	5μg PvCSP	IM	25,000 RAS (day 5)	IV	5000 WT SPZ (week 8)	4 days	0/5	0%		
10	25,000 RAS**	IV	25,000 RAS (day 5)	IV	5000 WT SPZ (week 8)	4 days	0/5	0%		
10	5ug PyCSP	IM	5µg PyCSP	IV	5000 WT SPZ (week 8)	4 days	0/5	0%		
10	–	–	–	–	5000 WT SPZ (week 8)	4 days	0/5	0%		

*Partially protected mice were rechallenged 6 weeks post-challenge.

**Mice were injected with RAS as priming dose.

To assess efficacy, immunized mice were challenged 3 weeks post-boost with an IV injection of 1000 wildtype (WT) Py 17XNL spz freshly dissected from mosquito salivary glands (Table 1) while the LION/repRNA-PfCS and LION/repRNA-PvCS prime-boost vaccines were used as irrelevant repRNA controls cohorts. None of the prime-boost cohorts, including the repRNA-PyCS vaccination-alone cohort, were protected against Py wild-type spz challenge in mice (Fig. 2c). However, ELISA at the endpoint (post-challenge, noted as “term” at day 48) showed that the antibody levels were recalled by the challenge dose of WT spz (*p* < 0.0001 relative to naïves, Fig. 2b). These repRNA-PyCS data show the consistency of the antibody responses induced by this repRNA platform, though full protection was not conferred by this homologous approach.

### Superior T-cell immunogenicity of repRNA-PyCS over gene-gun DNA priming in BALB/cJ mice

To assess T-cell responses to LION/repRNA-PyCS and evaluate this candidate as a potential priming dose in prime-and-trap vaccination, additional cohorts of BALB/cJ mice were immunized with a single IM injection and responses were assessed by splenocyte IFNγ ELISPOTs four weeks later (Fig. 2d). Responses were compared to splenocytes from mice immunized with a single repRNA-PfCS dose or DNA encoding PyCS administered by gene gun (gg DNA-PyCS), as used for priming in the first-generation prime-and-trap vaccine using RAS^32,33^. As expected, naïve mice and mice primed with the control LION/repRNA-PfCS did not recognize the PyCS epitope, as assessed by ELISPOT. In comparison, the LION/repRNA-PyCS vaccine elicited over tenfold more IFNγ-producing T cells than gene-gun DNA-PyCS vaccination (*p* = 0.0085, Fig. 2d), indicating robust priming of CD8 + T cells following a single LION/repRNA-PyCS injection.

### Immunogenicity of the accelerated prime-and-trap repRNA-PyCS-RAS vaccination in BALB/cJ mice

**Fig. 3:**
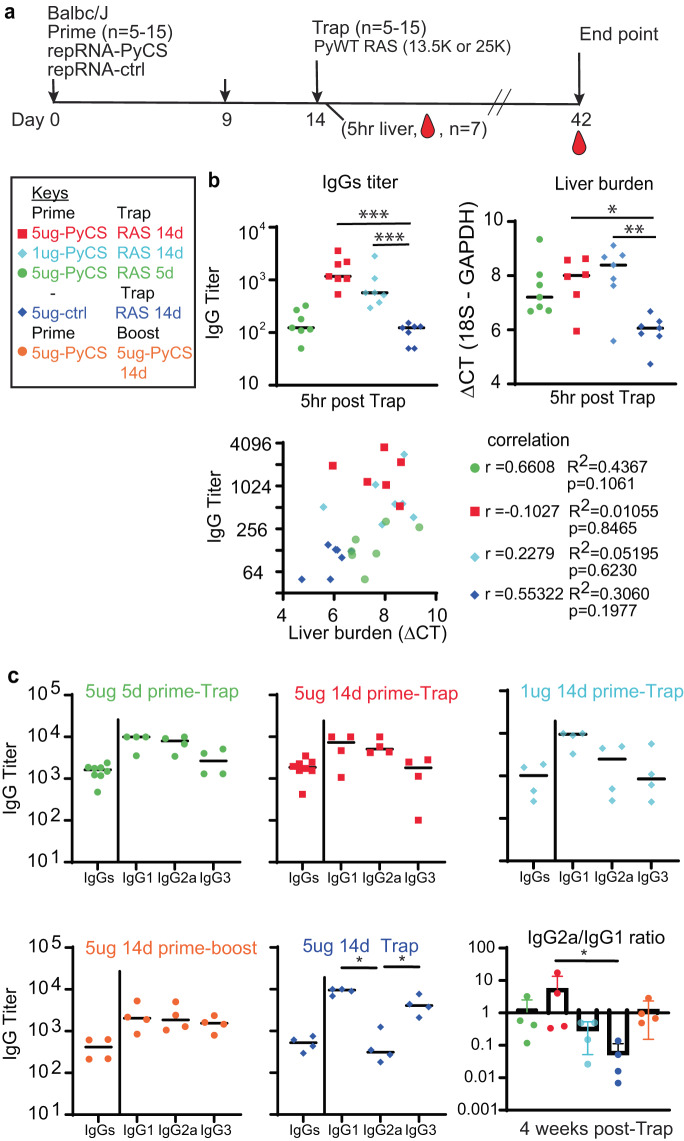
Immunogenicity of accelerated prime-and-trap immunization regimens in BALB/cJ mice. **a** Immunization schedule. 5ug 5-day (green data) or 5ug 14-day (red data) or 1ug 14-day regimen of repRNA-PyCS (light blue data) prime followed by trap dose of 25,000 RAS, were tested in mice. Control cohorts are prime-boost repRNA-PyCS (orange data) or trap cohort (repRNA-PfCS +RAS, dark blue data). **b** Liver and serum from seven mice per cohort were harvested 5 h post-trap injection. Total IgG titer was analyzed by ELISA, while liver parasite burdens were analyzed by qPCR quantification. Liver-burden qPCR was evaluated using one-way ANOVA followed by Kruskal-Wallis test and Dunn’s multiple comparisons test (*p < 0.05, **p < 0.005). A lower ΔCT represents a higher parasite burden. Pearson correlation comparing the IgG titer and the liver burden for the four vaccine groups. For visualization purposes, results from all cohorts are displayed in the same graph, but each correlation coefficient was computed individually for each cohort. **c** Final-bleed sera were collected at endpoint, 4 weeks post-trap (day 42), to evaluate total IgG titer and IgG1, IgG2a, IgG3 subclasses for each cohort by ELISA. Ratio of IgG2a/IgG1 is indicated in bar graph. All others statistical analyses were performed using a Mann–Whitney test. The n value represents total number of mice tested per cohort, and the error bars represent SD of the mean in two independent assays. Each data point represents an individual mouse, and the bar represents the group mean.

To assess LION/repRNA-PyCS as the priming dose in prime-and-trap vaccination, cohorts of BALB/cJ mice were immunized with LION/repRNA-PyCS followed by PyRAS, along with control cohorts receiving two doses of homologous prime-boost repRNA-PyCS, or PyRAS trap-only (consisting either of an irrelevant repRNA-PfCS control followed by PyRAS or a single dose of PyRAS alone). Naïve animals were used as additional controls. Previous prime-and-trap vaccine studies^24,32^ employed a 28-day interval between doses and utilized 20,000 to 50,000 RAS for the trapping dose. To investigate whether the schedule could be accelerated with the LION/repRNA prime and 25,000 PyRAS as the trapping dose, mice were primed IM with repRNA-PyCS (1 μg or 5 μg) 14 or 5 days prior to a PyRAS trapping dose (Fig. 3a).

To assess CS-specific whole IgG levels and parasite burden post-trapping, we collected sera and livers from seven mice of each cohort within 5–6 h after the trapping dose. As expected, mice immunized with either 14-day regimen (1 μg or 5 μg) had higher antibody titers than mice immunized with a 5-day regimen of repRNA-PyCS (trapped with RAS) and the irrelevant repRNA-PfCS regimen-with-RAS cohort (Fig. 3b). The repRNA-PfCS-plus-RAS cohort and the 5-day cohort exhibit an inverse trend between parasite burden in the liver and high levels of IgG (Fig. 3b). These results suggest that anti-PyCS IgG generated beyond 14 days post-prime targeted the WO spz of the trapping dose, reducing their distribution to the liver target.

Next, to determine if a balanced or skewed IgG subclass response was induced by repRNA-PyCS, we measured circulating IgG subclasses four weeks post-trapping dose in a separate cohort of mice, using CS peptide ELISA (Fig. 3c). There was no significant difference between the IgG1, IgG2a, and IgG3 levels, or in the IgG2a/IgG1 ratio between cohorts immunized with repRNA-PyCS as the priming dose, indicating that the two-dose regimen induces a balanced Th1/Th2 antibody response. However, the cohort receiving RAS alone as the trapping dose (i.e, 14-day repRNA-PfCS and RAS, Fig. 3c, dark-blue data) exhibited a skewed Th2-type humoral immune response. Our results implicate IgG2a in addition to the IgG1 and IgG3 subclasses as substantial components of the humoral response, as opposed to the narrow IgG1-biased response observed after administration of a WO-based vaccine such as RAS.

**Fig. 4:**
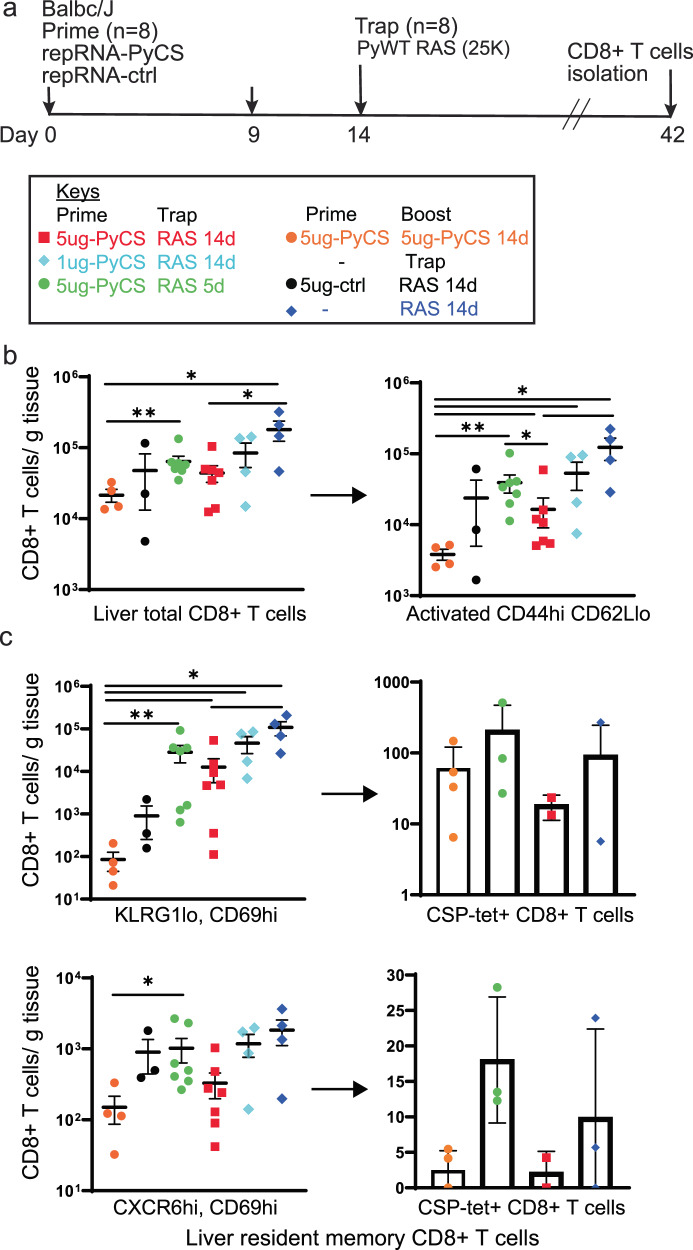
5-day prime-and-trap and trap-only (RAS) regimens of immunization induce higher-frequency liver Trm cells than 14-day prime-and-trap regimen. **a** Schedule of immunization. 5ug 5-day (green data) or 5ug 14-day (red data) or 1ug 14-day regimen of repRNA-PyCS (light blue data) prime followed by trap dose of 25,000 RAS (dark blue data), were tested in mice. Control cohorts are prime-boost repRNA-PyCS (orange data) or trap cohort (repRNA-PfCS +RAS, black data). **b** Flow cytometric total CD8^+^ T cells and activated (CD44hi/CD62Llo) CD8^+^ T cells in perfused livers 28 days after the Trap dose of 25,000 RAS. **c** Flow cytometric analysis of tetramer-stained, CS-specific CD8^+^ liver Trm cells (by CD69+ and either KLRG1lo (upper) or CXCR6+ (lower)). All error bars are SD of the mean. *p = 0.05, **p = 0.01, ***p = 0.001 by Mann–Whitney two-tailed test. The *n* value represents the total number of mice tested per cohort in two independent assays. Each data point represents an individual mouse and the bar represents the group mean, with error bars representing the standard error of the mean. Asterisks represent significance as determined by the non-parametric two-tailed Mann–Whitney U test (*p = 0.05, **p = 0.01, ***p = 0.001, ****p < 0.0001).

Finally, to assess CD8^+^ T-cell responses to LION/repRNA-PyCS prime-and-trap vaccination, mice were immunized under various regimens as described above (Fig. 4a). Livers were collected 28 days after the last immunization in two independent experiments, and lymphocytes were isolated and stained for flow cytometry as described previously^32^. Total CD8^+^ T cells and activated CD8^+^ T cells (CD44^hi^ CD62L^lo^) in the livers of prime-and-trap (5 μg 5-day, 5 μg 14-day, 1 μg 14-day) or trap-only (i.e., control prime-and-trap (5 μg 14-day) or 25,000 RAS) immunized mice were significantly higher than in the homologous prime-boost repRNA-PyCS-immunized mice (Fig. 4b). To determine if our prime-and-trap vaccine can generate CS-specific liver-resident Trm, CS-tetramer-labeled CD8^+^ T cells were identified by either CD69^+^/KLRG1lo or CD69^+^/CXCR6^+^ expression. Both populations of CS-specific liver-resident Trm were larger in the 5-day prime-and-trap cohort and the RAS-immunized mice than in the 14-day prime-and-trap immunized mice (Fig. 4c). This result is consistent with the observation that post-trap liver parasite burden is lower in the 14-day group, as shown above, resulting in reduced Trm generation.

### Efficacy of accelerated prime-and-trap repRNA-PyCS-RAS vaccination in BALB/cJ mice

**Fig. 5:**
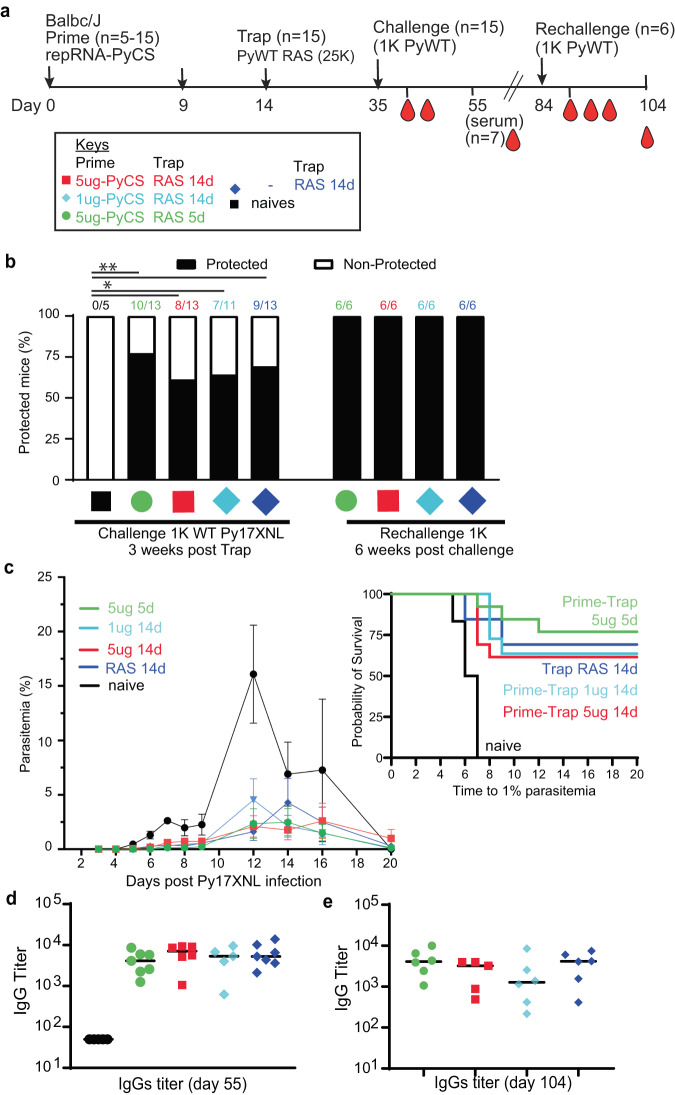
Efficacy of accelerated prime-and-trap immunization regimens in BALB/cJ mice. **a** Immunization schedule. Prime dose of 5 μg 5-day (green data) or 5 μg 14-day (red data) or 1 µg 14-day regimen of repRNA-PyCS (light blue data), followed by trap dose (dark blue data) of 25,000 RAS, were tested in mice. Three weeks later, mice were challenged intravenously with 1000 live spz isolated from infected mosquitos. Control cohorts are the trap cohort (RAS alone, dark blue data) and naïve cohort. Cohort showing partial protection were rechallenged with 1000 live spz six weeks later. **b** Protection post-challenge and re-challenge per cohort. Number of mice per cohort indicated above bar graph as protected/non-protected ratio. **c** Parasitemia post-challenge of immunized mice cohorts and patency curves (>1% parasitemia) of mice post-challenge. **d** Sera from seven mice per cohort were harvested 20 days post-challenge (day 55) and CS-specific IgG titers were analyzed by ELISA. **e** Final bleeds (six weeks post-rechallenge, day 104) of the four cohorts of mice showing full protection. The n value represents the total number of mice tested per cohort, in two or three independent assays. Each data point represents an individual mouse and the bar represents the group mean. All others statistical analyses were performed using a Mann–Whitney two-tailed test.

To assess whether the immune responses induced by prime-and-trap immunization can confer sterile immunity, cohorts of mice immunized with LION/repRNA-PyCS as prime dose followed by RAS for the trapping dose (as described above in Fig. 3) were challenged three weeks later with 1000 freshly prepared WT Py spz delivered IV (Fig. 5a, Table 1). Upon challenge, 66% of trap-only and 80% of prime-and-trap mice were sterilely protected (Fig. 5b; naïve mice were not protected). These results (66% vs 80%) are statistically indistinguishable. However, in the mice of the immunized cohorts that experienced breakthrough parasitemia, we saw a consistent 2- to 3-day delay in the onset of blood-stage parasitemia and a reduced peak load (Fig. 5c), indicating reduced liver burden. Two weeks post-challenge, sera were collected and total IgG levels were quantified by ELISA. The total IgG titer post-challenge was similar in all immunized cohorts (prime-and-trap vs trap alone), suggesting a recall of the CS-specific humoral immune response following spz challenge (Fig. 5d).

The four cohorts exhibiting partial sterile protection post-challenge were re-challenged 6 weeks after the first challenge (Table 1). All were protected (Fig. 5b) with high levels of circulating specific anti-CS IgG (Fig. 5e). On the whole, the data from these replicate experiments are consistent with previous observations of limited protection after a single dose of PyRAS, indicating that the prime-and-trap strategy is important for making the most of the LION/repRNA-primed responses.

### Prime-and-trap vaccination improves protection against more stringent challenge in BALB/cJ mice over trap alone

**Fig. 6:**
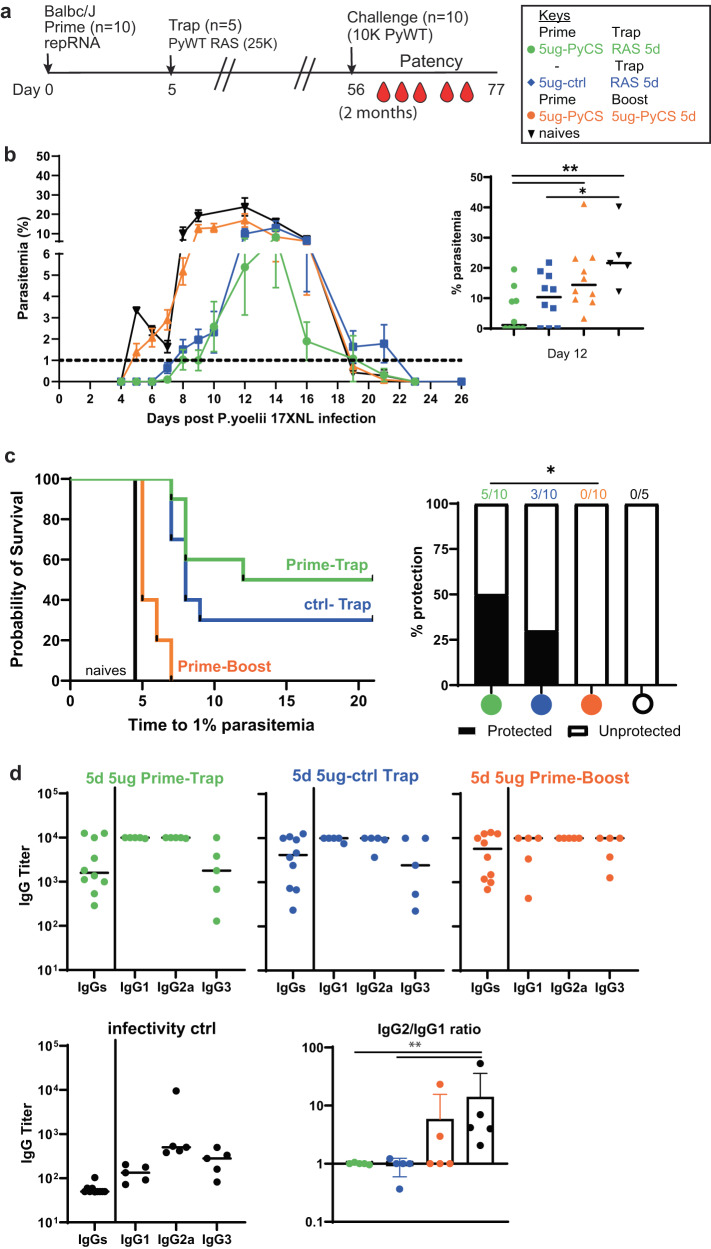
Prime-and-trap vaccine improves protection against stringent challenge in BALB/cJ mice. **a** Schedule of immunization. Prime-and-trap vaccine composed of 5ug 5-day regimen of prime with repRNA-PyCS (green data) followed by trap dose of 25,000 RAS. Control cohort is a 5 μg 5-day prime-boost repRNA-PyCS (orange data) or a trap cohort (repRNA-PfCS +RAS, dark blue data). Two months later, mice were challenged intravenously with 10,000 live spz isolated from infected mosquitos. **b** Parasitemia post-challenge of all cohorts including the naive cohort. Emphasis indicates the parasitemia peak at day 12, each dot representing a mouse, and the bar is the mean of the cohort. **c** Patency curves of mice post-challenge per cohort. Number of mice per cohort indicated above bar graph as protected/non-protected ratio. ****p < 0.0001 by Fisher exact test. **d** Final-bleed sera were collected at endpoint (day 77) to evaluate total IgG titer and IgG1, IgG2a, IgG3 subclasses from each cohort by ELISA. Ratio of IgG2a/IgG1 is indicated in bar graph. The n value represents the total number of mice tested per cohort, and the error bars represent SD of the mean in two independent experiments. Each data point represents an individual mouse and the bar represents the group mean. All statistical analyses were performed using a Mann–Whitney two-tailed test. *p = 0.05, **p = 0.01, ***p = 0.001 unless specified in panel.

To determine if prime-and-trap vaccination can protect against a more stringent challenge, the efficacy of a 2-dose 5-day prime-and-trap immunization was compared to a trap-only regimen (irrelevant prime repRNA-PfCS + RAS). BALB/cJ mice were vaccinated with 5 μg repRNA-PyCS or the irrelevant rep-RNA-PfCS once, followed by a dose of 25,000 RAS 5 days later (Table 1). As a control cohort, mice were immunized 5 days apart with 2 injections of 5 μg repRNA-PyCS (homologous prime-boost). Mice were challenged with 10,000 Py WT spz at 8 weeks post-trap (Fig. 6a, b). We observed results similar to those previously reported, i.e., the homologous prime-boost repRNA-PyCS cohort showed no protection following challenge (Fig. 6b, c, orange data), and was indistinguishable from our infectivity-control cohort (Fig. 6b, c, black data). Following this 8-week challenge with a high challenge dose, the efficacy of a trap-only (RAS) vaccine was just 30% (Fig. 6c, blue data) and was 50% with a prime-and-trap vaccine (Fig. 6c, green data). However, only the 5-day regimen prime-and-trap yielded a moderate but significantly (*p* = 0.0325) higher level of sterile protection than prime-boost immunization. After challenge, high titers of anti-CS IgG were detected by ELISA in all prime-and-trap cohorts and no difference was observed in IgG subclasses among the three repRNA-immunized cohorts (Fig. 6d).

### Prime-and-trap repRNA-PyCS-RAS vaccination does not elicit sterile protection in C57Bl6 mice

To further demonstrate the importance of CS-specific CD8 + T cells for protection of BALB/cJ mice, C57BL/6 mice (MHC H2b) that are unable to present the protective MHC H2-Kd-restricted epitope SYVPSAEQI were immunized and challenged. C57BL/6 mice do not express the MHC-I allele needed to present this CS-specific epitope in infected hepatocytes. C57BL/6 mice were immunized according to a 5-day regimen with 5 μg repRNA-PyCS and 25,000 RAS (Supplementary Fig. 3A, Table 1). The prime-and-trap cohort (repRNA-PyCS + RAS) was compared to a prime-boost repRNA-PyCS cohort, a trap cohort (repRNA-PvCS + RAS), a double-trap (RAS + RAS) cohort, and an infectivity-control cohort. At day 35 after the second immunization, sera were collected and analyzed by ELISA against the PyCS peptide (Supplementary Fig. 3B). Both trap and prime-boost cohorts had significantly lower IgG titers than the double-RAS and prime-and-trap cohorts, which exhibited equivalent strong anti-CS antibody titers (Supplementary Fig. 3b).

These animals were then challenged 8 weeks later by an IV dose of 5000 infectious WT Py spz. As anticipated, none of the C57BL/6 mice cohort had sterile protective immunity following challenge (Supplementary Fig. 3C), but the trap, double-trap, and prime-and-trap animals experienced a delay in the onset of parasitemia compared to the infectivity control and prime-boost cohorts (Supplementary Fig. 3C). Moreover, relative to the infectivity control cohort (Supplementary Fig. 3D, black data), animals immunized with either prime-and-trap (Supplementary Fig. 3D, green data) or double trap (Supplementary Fig. 3D, blue data) showed significantly greater control of the parasitemia (particularly at the day of peak infection) than the cohorts immunized with either irrelevant repRNA-PvCS and RAS (Supplementary Fig. 3D, purple data) or prime-boost double repRNA (Supplementary Fig. 3D, orange data). Interestingly, while the antibody response to the homologous prime-boost repRNA immunization was biased toward IgG2c (Supplementary Fig. 3E, orange data), all other regimens induced more balanced IgG2c:IgG1 ratios (Supplementary Fig. 3E, prime-and-trap in green, prime control-and-trap in purple and double trap (RAS) in blue).

### Sterile protection following same-day prime-and-trap repRNA-PyCS-RAS vaccination

**Fig. 7:**
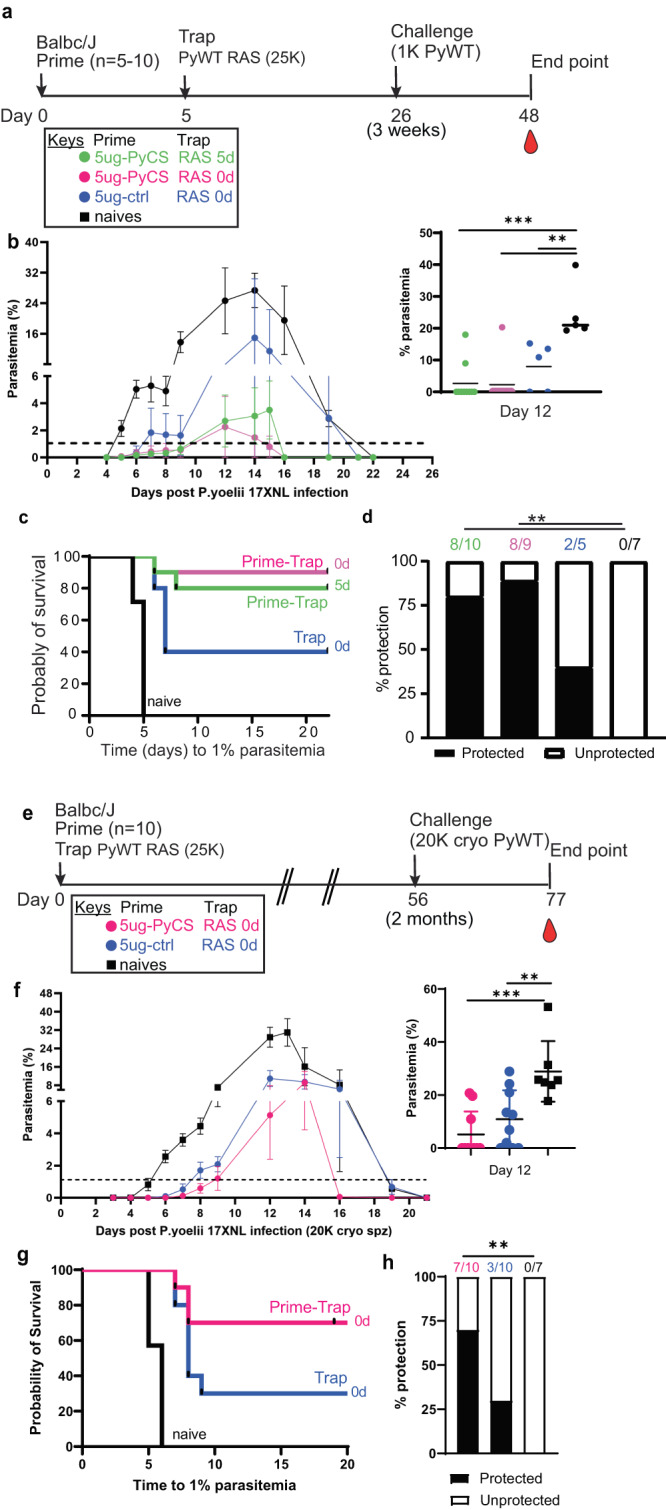
Immunogenicity and efficacy of a same-day prime-and-trap vaccine in BALB/cJ mice. **a** Schedule of immunization. Prime-and-trap vaccine consisted of a 5ug 5-day regimen (green data) or 5ug same-day interval (pink data) of prime with repRNA-PyCS followed by trap dose of 25,000 RAS. Control cohort is 5 μg same-day interval of a trap cohort (repRNA-PfCS +RAS, dark blue data). Three weeks later, mice were challenged intravenously with 1000 live spz isolated from infected mosquitos. **b** Parasitemia post-challenge of all cohorts including the naive cohort (black data). **c** Patency curves (>1% parasitemia) of mice post-challenge. **d** Protection post-challenge per cohort. Number of protected mice per cohort indicated above bar graph. **e** Schedule of immunization of a same-day prime-and-trap vaccine (5 μg, 0-day regimen) with trap dose of 20,000 cryopreserved spz administered IM and IV, respectively, on the same day. Control cohort is naive. Two months later, mice were challenged intravenously with 20,000 cryopreserved spz isolated from infected mosquitos. **f** Patency curves (>1% parasitemia) of mice post-challenge. **g** Patency curves (>1% parasitemia) and protection (h) post-challenge per cohort. Number of protected mice per cohort indicated above bar graph. ****p < 0.0001 by Fisher exact test. The *n* value represents the total number of mice tested per cohort, in two independent experiments. All other statistical analyses were performed using a Mann–Whitney two-tailed test *p = 0.05, **p = 0.01, ***p = 0.001.

As described above, accelerating the prime-and-trap vaccination from a 14-day to a 5-day immunization schedule reduced levels of circulating CS-specific antibodies (Fig. 3b) and improved numbers of CS+ liver Trm (Fig. 5), while retaining strong efficacy (Fig. 4e). We next compared the protective efficacy in BALB/cJ mice of same-day and 5-day prime-and-trap regimens. Mice were vaccinated with 5 μg repRNA-PyCS and 25,000 RAS and challenged 3 weeks later by an IV dose of 1000 freshly dissected infectious WT Py spz (Fig. 7a, Table 1). These cohorts were compared to a trap-alone (repRNA-PfCS + 25,000 RAS) cohort and an infectivity-control cohort. While both the trap cohort (3/5 mice) and control cohort (7/7 mice) rapidly developed high levels of parasitemia, parasitemia appeared in only 2/10 and 1/9 of the 5-day and 0-day prime-and-trap cohorts, respectively (Fig. 7b–d). In the prime-and-trap cohorts, parasitemia appeared 7–8 days after infection and was cleared by day 16 (Fig. 7b), whereas in both control cohorts the parasitemia appeared 4–5 days post-infection, persisted for 15 days, and was cleared at day 22 post-challenge as anticipated.

To determine if a same-day prime-and-trap vaccine can protect against a more stringent challenge, a cohort was immunized with 5 μg repRNA-PyCS and 25,000 RAS on the same day and challenged two months later with 20,000 cryopreserved WT Py spz (previously frozen in-house as described^49^ and thawed and injected within 30 minutes) (Fig. 7e). All the infectivity-control cohorts consistently developed parasitemia by day 5 to 6, while the prime-and-trap and trap cohorts showed a 2-3-day delay (Fig. 7f–g). Upon challenge two months post-immunization, 7/10 (70%) of the prime-and-trap immunized mice showed sterile immunity, whereas 3/10 (30%) of the trap cohort were protected (Fig. 7g, h). These results support the feasibility of an accelerated prime-and-trap immunization with 2 concurrent injections via IM and IV routes, respectively.

## Discussion

An effective malaria vaccine will save many lives and lift the global burden of this disease. Among the next-generation approaches that build on the recent success of vaccines in COVID-19 prevention are mRNA vaccines delivered with nanoparticles^34,35^. Our prime-and-trap approach combines two strategies that exhibit modest efficacy individually but confer promising levels of protection when combined. This approach also has key logistical advantages over other current candidates. We summarize all the mouse immunization and challenge studies performed in this manuscript with Py wild-type parasites in Table 1. We have shown that a homologous LION/repRNA-PyCS vaccine (prime-boost) is highly immunogenic at all doses evaluated, eliciting strong antibody responses when given in a 2-dose immunization regimen 2 weeks apart. Antibody levels are modest after the priming dose but increase subsequent to the booster dose. We have also demonstrated that homologous LION/repRNA-CS vaccination alone was insufficient to prevent blood-stage infection and did not provide protection in mice. To overcome these issues and enhance the practicability of vaccinating people in low-resource environments, our efforts have focused on minimizing the interval between injections, lowering dosages, and simplifying storage and preparation of the agents. We believe an effective vaccine based on a strategy such as that described herein, administered in a single clinic visit, will enhance the logistics of the vaccination schedule and reduce the cost of goods.

Our results suggest that the priming dose of LION/repRNA-PyCS is highly immunogenic. We observed induction of a strong humoral response in BALB/cJ and C57Bl/6 mice when they were immunized on the 14-day, 5-day, or same-day regimens. We also observed a strain-specific CD8 + T-cell response in BALB/cJ mice. There was a 2-3-day delay in the onset of blood-stage parasitemia compared to the control groups, which indicates a reduction in liver burden. Our prime-and-trap approach has the advantage of inducing both humoral and cellular immunities without compromising the generation of protective liver-resident CD8^+^ T cells. Since the effectiveness of the RAS trapping dose may be affected by the presence of CS-specific antibodies, future work will include evaluation of how our vaccine performs in the context of infection-induced or vaccine-induced immunity.

Previous studies have shown that RAS vaccines can induce liver CD8^+^ Trm cells in animal models, but they can only achieve high levels of sterile protection in humans with three or more IV doses^20,50,51^. The requirement for several IV doses compounds the time, cost, and logistical problems inherent to WO vaccines^27,28^. Others studies employing later-arresting GAP vaccines^24,50,52^ have shown that expressing more antigens leads to improved protection over early-arresting GAP or RAS^16,53^. Likewise, immunization with wild-type (WT) WO spz administered under blood-stage drug chemoprophylaxis (known as CPS, (PfSPZ-CVac)^22,54^) allows full liver-stage development and can induce high levels of protection at reduced doses, although CPS involves co-administration of drugs with the vaccine, presenting practical and regulatory challenges^22^. These well-established immunizations with WO SPZ RAS^55,56^, GAP^16,24,52,57^, or PfSPZ-CVac^22,54^ are at least in part reliant on the generation of liver-resident memory CD8^+^ T cell (Trm) responses^19,31,58–60^.

In the present study, we show the contribution of CS-specific liver-resident memory CD8^+^ T cells induced by our prime-and-trap regime with LION/repRNA-PyCSP. Given that the initial dose of RAS administered to naïve individuals appears to be the most immunogenic and effective in inducing liver Trm cells, a vaccination strategy employing a single dose of RAS^32^ may be the most efficient and cost-effective means of using this valuable resource.

Our approach of combining repRNA and LION has several advantages over mRNA-LNP vaccines: a reduced number of doses (2), the use of a single dose of the WO irradiated spz, a shorter (5-day or same-day) administration schedule, and simpler logistics^34^. Nanoparticle formulations as carriers for nucleic acid delivery make it possible to optimize for specific target cells and tissue types. The balance of tissue targeting can be shifted by changing the nanoparticle composition and route of delivery (oral, subcutaneous, or IV)^61^. We will initially focus future work on lyophilization of our repRNA malaria vaccines to eliminate cold-chain requirements prior to reconstitution, and secondly on direct targeting of the liver, in the hope of replacing spz administration with a more practical and cost-effective nanoparticle formulation.

Our findings reinforce the concept that CS remains one of the most immunodominant and protective antigens expressed by spz^62^, and that its CD8 + T cell epitope is probably involved in the protective effect against parasites in the liver of the BALB/cJ mice following RAS immunization. Indeed, when C57BL/6 mice were challenged, sterile protection was not achieved, since the SYIPSAEKI immunodominant epitope cannot be presented by the C57BL/6 MHCI. Although the CS protein still appears to be the best vaccine candidate antigen it is not clear that any single antigen will confer the robust and durable protection needed to eradicate malaria. Given the genetic diversity among parasite strains and the many variables in physiology, environment, and logistical capabilities involved in a broad vaccination campaign, it may be necessary to target multiple antigens from various stages of the parasite life cycle to eliminate malaria. We are currently evaluating the immunogenicity of repRNA presenting multiples antigens from various stages of the parasite life-cycle.

In summary, this replicating RNA/LION vaccine for malaria to show greater than 70% sterile protection in mice. We have demonstrated a heterologous vaccination approach that concurrently induces humoral and T-cell immunities, using repRNA-CS formulated with LION nanoparticles and PyRAS targeting the liver. These promising preliminary data suggest that sterile protection in mice may be achievable by making further rational refinements to this approach. This prime-and-trap approach is currently being broadened in mouse studies to include antigens from other stages of malaria, which will be the focus of a future manuscript. Our prime-and-trap approach is also being tested in non-human primate models, potentially leading to immunogenicity and efficacy studies in a CHMI trial in the near future.

## Methods

### Vaccine design

The antigen chosen for our vaccine design is the full-length codon-optimized circumsporozoite (CS) protein (PyCS XP_728216.3; PfCS XP_001351122.1; PvCS VUZ95499.1) from *Plasmodium*, as described in detail in Supplementary Fig. 1. The coding sequences of the full-length CS protein from Pf and Pv were utilized as irrelevant RNA controls. The PyCS antigen has a CD8^+^ T-cell epitope that is immunodominant and protective in the H2-K^d^ (BALB/cJ)-restricted genetic background.

### RNA production and LION formulation

Full-length CS coding sequences from *Pf*, *Pv*, and *Py* were cloned separately into a Venezuelan equine encephalitis (VEE) replicon vector (pT7-VEE-Rep). In vitro transcription was performed at 34 °C using a T7 MEGAscript T7 Transcription kit (Invitrogen). RNA was purified via lithium-chloride precipitation, followed by capping with a capping kit (New England Biolabs) as described^42^. RNA was further purified and stored at -80° C until use. Denatured repRNAs were verified by electrophoresis in a 1% agarose gel. Briefly, 2 μg of each repRNA was denatured by glyoxal treatment (NothernMax-Gly, AM8551, ThermoFisher), and run in an agarose gel with NorthernMax-Gly gel prep/running buffer (AM8678, ThermoFisher). Ethidium bromide was premixed into the running buffer and the gel image was analyzed by a Biorad gel docXR+ Imaging system.

To protect the RNA replicons from degradation, we combined them with LION nanoparticles obtained from HDT Bio^42^. LION nanoparticles consist of a hydrophobic squalene oil core stabilized with Tween 80, Span 60, and the cationic lipid DOTAP. The oil (squalene, span 60 and DOTAP) and aqueous (Tween 80 in 10 mM sodium citrate) phases were homogenized using an L5M-A high-shear mixer (Silverson) and further processed by passaging through a microfluidizer to achieve an average hydrodynamic diameter of 60 nm and polydispersity index of 0.2 by dynamic light scattering. The microfluidized LION was terminally filtered with a 200-nm pore-size polyethersulfone filter and stored at 2° to 8 °C.

### Cells lines

To qualify the vaccine candidates in vitro, BHK cells (American Type Culture Collection) were transfected with repRNA or mock-transfected using OptiMEM (Gibco) and Expifectamine transfection kit (ThermoFisher). Cells were scraped off and lysed with RIPA buffer 24–48 h later, and lysates were analyzed by SDS–polyacrylamide gel electrophoresis and Western blot.

### Western blots

Cells lysates were analyzed by Western blot after transfer to nitrocellulose membranes. For detection, anti–rabbit polyclonal anti-CSP (Py, Pf, or Pv) antibodies (Pocono) were used (1/1000) followed by goat anti-rabbit IgG (H + L) alkaline phosphatase-linked secondary antibody (Invitrogen, T2191) (1/10,000).

### Ethics statement

Animal studies were performed according to the regulations of the Institutional Animal Care and Use Committee of Bloodworks Northwest, and approval was obtained from this committee. Our studies meet the standards of the Guide for the Care and Use of Laboratory Animals and applicable Bloodworks Northwest policies and procedures. Bloodworks Northwest has an approved Animal Welfare Assurance (#A4659-01, D16-00862) on file with the NIH Office of Laboratory Animal Welfare (OLAW).

### Mice

Female BALB/cJ and C57Bl/6 (B6) mice, six to eight weeks old, were purchased from The Jackson Laboratories (Bar Harbor, ME, USA). Mice were maintained under pathogen-free conditions in animal facilities and were fed autoclaved food ad libitum. Mice were housed and cared for in standard IACUC-approved animal facilities at Bloodworks Northwest and used in compliance with IACUC-approved protocol 5285-01, which adheres to the NIH Office of Laboratory Animal Welfare standards. Mice were anesthetized using isoflurane and final bleed was collected by cardiac puncture, before been cervically dislocated inside a biosafety cabinet. For organs collection, mice were then doused with 70% ethanol and opened using sterile scissors and forceps. All methods are reported in accordance with ARRIVE guidelines.

### LION/repRNA vaccination

For all LION/repRNA vaccines, 5 μg of RNA was mixed with LION at a nitrogen:phosphate (N:P) ratio of 15 and injected IM into mice using a total of 50 µl (25 µl in each leg). A two-vial formulation method was performed as described^42^.

To determine the immunogenicity of homologous prime-boost LION/repRNA-CS vaccination with single or dual CS antigens, mice were immunized with an IM prime of 5 µg of LION/repRNA, followed by a homologous boost 14 days later. The mice received a LION/repRNA vaccine with either one of the antigens (PyCS or PfCS; 5 μg per antigen) or a combination of two antigens (PyCS and PfCS, 2.5 μg per antigen) (Supplementary Fig. 2A).

Other immunization prime-and-trap protocols and timelines are presented in each respective figure.

### Sporozoite isolation, vaccination, and challenge

Wild-type Py (17XNL strain) spz were prepared by cyclical transmission in BALB/cJ mice and *Anopheles stephensi* mosquitoes at the Seattle Children’s Center for Global Infectious Disease Research Insectary (Seattle, WA, USA). Female 6- to 8-week-old Swiss Webster (SW) mice were injected with blood-stage Py 17XNL WT parasites to begin the growth cycle and used to feed female *Anopheles stephensi* mosquitoes. At day 15 after blood meal, salivary-gland spz were isolated and harvested as previously described^63^. RAS were generated by exposure to 10,000 rads using an X-ray irradiator (Rad-Source, Suwanee, GA, USA). RAS were resuspended in 100 μL Schneider and administered to the mice through tail-vein injection. Infectious spz for challenge were prepared in an equivalent manner but without irradiation. All experimental and control mice were challenged with live *Py* 17XNL spz. A summary of vaccination and challenge experiments is included in Table 1.

### Liver lymphocyte isolation and flow cytometry

Livers were perfused with 10 ml PBS/2 mM EDTA by injection into the portal vein, with outlet drainage via the inferior vena cava, and mashed into a single-cell suspension. Intrahepatic lymphocytes were isolated as previously described^32,33^. Final pellets were resuspended in 150 μl 1x MACs buffer and transferred to a 96-well plate for blocking and staining prior to flow cytometry. All antibody characterizations and flow-cytometry analyses were performed as previously described^28,33^, using a live/dead dye (Zombie NIR Fixable Viability Kit, BioLegend) to enable exclusion of dead cells from downstream analysis. In brief, liver lymphocytes were treated with an Fc block (anti-CD16/32, clone 2.4G2; BD Biosciences) and live/dead dye for 30 min, stained for 45 min (with antibody cocktail as described^33^), and fixed for 20 min (Cytofix/Cytoperm reagent; BD Biosciences). Cells were gated for CD8 + T cells (CD3e + , B220-, CD4-), CD44hi by CD62Llo, then assessed by either KLRG1lo by CD69hi or by CXCR6hi by CD69hi. Antigen specificity was then assessed by PyCSP-tetramer (SYVPSAEQI-specific H2-Kd tetramer, National Institutes of Health Tetramer Core) conjugated to streptavidin-allophycocyanin (ProZyme) per standard protocols. Cell count per gram of tissue was calculated based on a known concentration of counting beads per sample to normalize data. Flow cytometry was performed on an LSR II (BD Biosciences), and data analysed with FlowJo version 10.7.1 (BD Biosciences).

### Ex vivo IFNγ ELISPOT

Spleens were harvested and splenocytes separated from BALB/cJ mice 28 days post-immunization. A total of 1x10E5 splenocytes were combined with SYVPSAEQI peptide (1 mg/ml final) (Genemed Synthesis) for murine IFNγ ELISPOT (eBioscience), cultured for 18 h at 37 °C, and developed following manufacturer guidelines. The percentage of antigen-specific T cells was calculated based on the spot-forming units counted in each well divided by the total number of splenocytes applied to each well.

### Blood stage

Breakthrough to blood-stage patency was assessed by Giemsa-stained thin blood smear starting at day 4 after challenge and ending at day 21, at which time a negative smear was attributed to complete protection.

Mice immunized with Pf- or Pv-repRNA-CS and challenged with live Py spz were used as controls. Sterile protection was defined as being blood-smear negative. The Kaplan-Meier curves illustrate the time to developing parasitemia during days 4–21 after challenge with 17XNL strain Py live spz.

### qRT-PCR

Liver burden was detected by qRT-PCR from harvested livers 44 h post-challenge^32,64^. Total RNA was extracted from Py-infected livers using TRIzol reagent (Thermo Fisher Scientific) and treated with Turbo DNase (Ambion). cDNA synthesis was performed using a SuperScript III Platinum two-step qRT-PCR kit (Thermo Fisher Scientific). Specific PCR primers (as listed below) were used to amplify *Py* 18 S rRNA and GAPDH (housekeeping) gene from cDNA derived from mouse liver. The primers used for amplification of 18 S rRNA from cDNA were 18S-fwd: (GGGGATTGGTTTTGACGTTTTTGCG) and 18S-rev: (AAGCATTAAATAAAGCGAATACATCCTTAT). Mouse GAPDH was amplified with cDNA using gapdh-fwd: (CCTCAACTACATGGTTTACAT) and gapdh-rev: (GCTCCTGGAAGATGGTGATG) primers. All qRT-PCR amplification cycles were performed at 95 °C for 30 s (DNA denaturation) and 60 °C for 4 min (primer annealing and extension). Samples are run in triplicate. Results are expressed as the difference ΔCT in threshold cycle number between the average of CT value of the Py 18 S parasite gene and the average of CT value of the GAPDH house-keeping gene, so a high ΔCT represents a low parasite burden.

### ELISA

MaxiSorp plates were coated overnight with 100 μL CS peptide (PyCS QGPGAPQGPGAPQGPGAPQGPGAP, PfCS PNANPNANPNANPNANPNAN, Genscript) at 1μg/ml in PBS 4 °C. Plates were then washed with PBS + 0.05% Tween 20 (PBS-T) and blocked with 1% BSA in PBS-T for 2 h at RT. Murine serum samples were plated at a dilution of 1:50 in PBS-T + 0.1% BSA, serially titrated 1:3 for 6 wells, and incubated for 2 h at RT or overnight at 4 °C. Following washing steps, plates were incubated with goat anti-mouse secondary antibodies, HRP diluted 1:5000 (62-6520, Thermo Fisher Scientific) in PBS-T + 0.1% BSA for 1 hr at RT. After a second wash, 100 μL TMB (substrate 95059-286, VWR) was added per well and incubated 5–10 min before stopping with 50 μL 1 N sulfuric acid.

### Statistical analysis

Comparisons of ELISA groups or flow-cytometry cell counts were done using the non-parametric two-tailed Mann–Whitney U test (*p = 0.05, **p = 0.01, ***p = 0.001, ****p < 0.0001). ELISPOT assay comparisons were done by unpaired, two-tailed Student’s *t* tests. Statistical significance between groups of mice for their liver burden qRT-PCR was evaluated using one-way ANOVA followed by the Kruskal-Wallis test and Dunn’s multiple-comparisons test (* *p* < 0.05, ** *p*< 0.005). Protection data were evaluated using Fisher’s exact test. All groups were compared against the prime-boost cohort or the trap-alone cohort (repRNA-PfCS or repRNA-PvCS for priming, and RAS for trapping dose; **** *p* < 0.0001). Error bars are SEM of the mean with individual mouse samples shown. Statistical significance was defined as *p* < 0.05 using Prism Graph-Pad 9.4.1 Software (San Diego, CA).

### Reporting summary

Further information on research design is available in the Nature Research Reporting Summary linked to this article.

### Supplementary information

Supplemental material

REPORTING SUMMARY

## Data Availability

The authors declare that all data generated or analyzed during this study are included in this study are available within the main and supplemental figures. The data supporting this study’s findings are available from the corresponding authors upon reasonable request.
