# Supplementary material for: Accelerated prime-and-trap vaccine regimen in mice using repRNA-based CSP malaria vaccine
Source: NPJ Vaccines. 2024 Jan 10;9:12. doi: 10.1038/s41541-023-00799-4 (PMC10781674; doi:10.1038/s41541-023-00799-4)
Supplement: Supplementary file 1 — Supplemental material [file 41541_2023_799_MOESM1_ESM.docx]

### SUPPLEMENTAL INFORMATION

### Accelerated prime-and-trap vaccine regimen in mice using repRNA-based CSP malaria vaccine

### Zach MacMillen^1^, Kiara Hatzakis^1^, Adrian Simpson^2^, Melanie J. Shears^3^, Felicia Watson^3^, Jesse H. Erasmus^2^, Amit P. Khandhar^2^, Brandon Wilder^4^, Sean C. Murphy^3^, Steven G. Reed^2^, James W. Davie^1^_,_ Marion Avril^1*^

### MalarVx, Inc 1551 Eastlake Ave E, Suite 100, Seattle WA 98102

### HDT Bio, 1616 Eastlake Ave E, Suite 280, Seattle WA 98102

### University of Washington, Department of Laboratory Medicine and Pathology, 750 Republican St., F870, Seattle, WA 98109

### Vaccine & Gene Therapy Institute, Oregon Health & Science University, Building 1, Room 2220, 505 NW 185th Ave, Beaverton, OR 97006

### *Correspondence [mavril@malarvx.com](mailto:jdavie@malarvx.com)

**
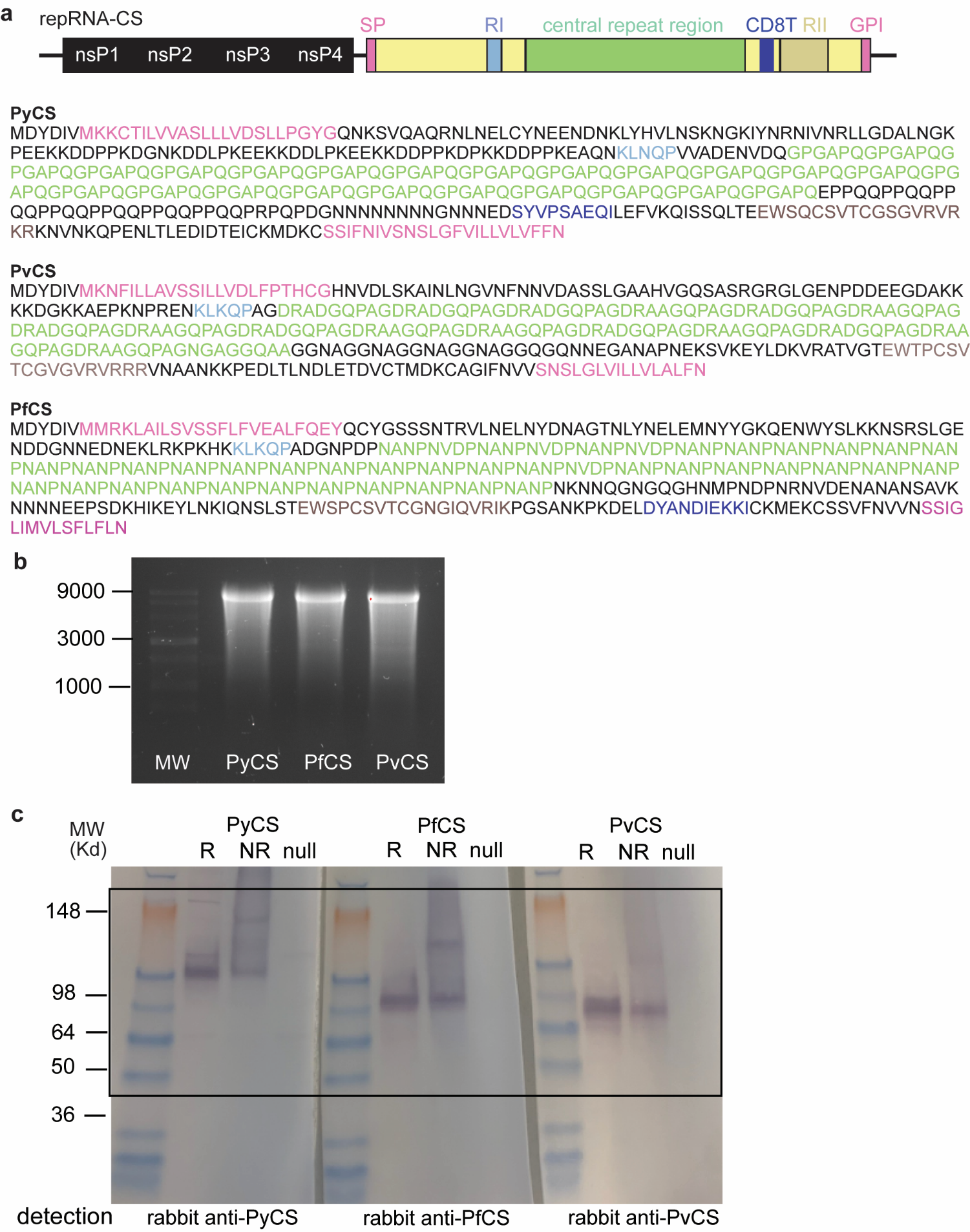
**

**Supplemental Fig 1. CS replicon and protein sequences.** a) Schematic of the replicon encoding the full length of either *P. yoelii*, *P. vivax,* or *P. falciparum* circumsporozoite (CS) protein including the signal peptide (SP) at the N-terminus, the region I (RI), the entire central repeat region, the CD8^+^ T cell epitope, the region II (RII) up to the GPI anchor signal in C-terminal. The replicon was cloned into an alphavirus replicon encoding the four nonstructural protein (nsP1 to nsP4) genes of the Venezuelan equine encephalitis virus (VEE) strain TC-83. For each CS cloned into the replicon, the full protein sequence is indicated. b) Northern denaturing agarose gel of repRNA-PyCS, -PfCS, and -PvCS. c) Uncropped western blot from Fig 1a showing protein expression.

**
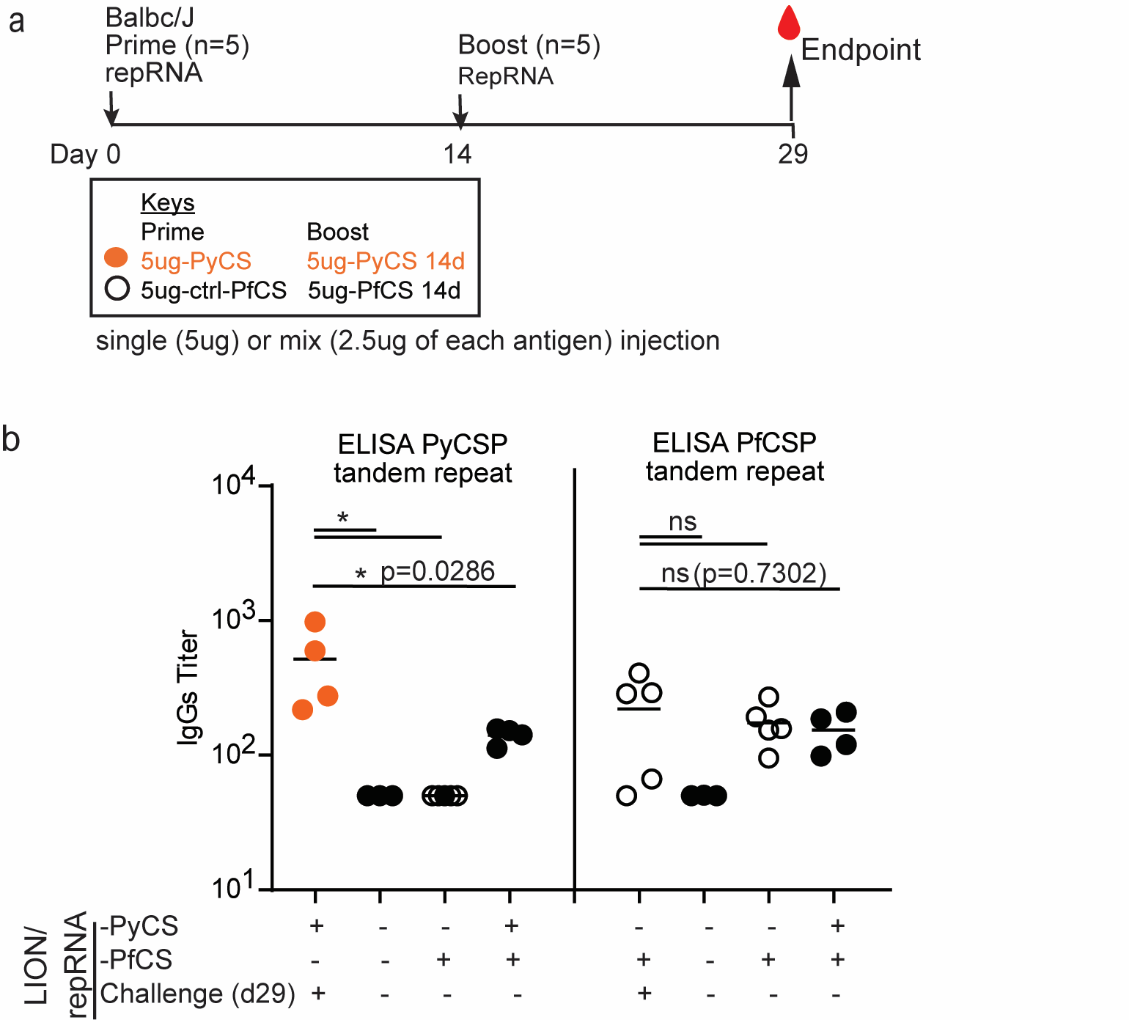
**

**Supplemental Fig 2. Immunogenicity in BALB/cJ of repRNA-CS formulated with LION.** a) Immunization schedule for the 14-day Prime-Boost with either LION/repRNA-PyCS or -PfCS. used as control replicon. n=5 mice per cohort in one experiment. b) Mice were either immunized with a single antigen (5μg each) or two antigens mixed (2.5μg each). Final bleeds were collected two weeks post-boost at endpoint and immune responses were analyzed by ELISA against their corresponding CS tandem repeat regions.. Each data point represents an individual mouse, and the bar represents the group mean. Asterisks represent significance as determined by non-parametric two-tailed Mann–Whitney U test (*p=0.05, **p=0.01, ***p=0.001, ****p<0.0001).

**
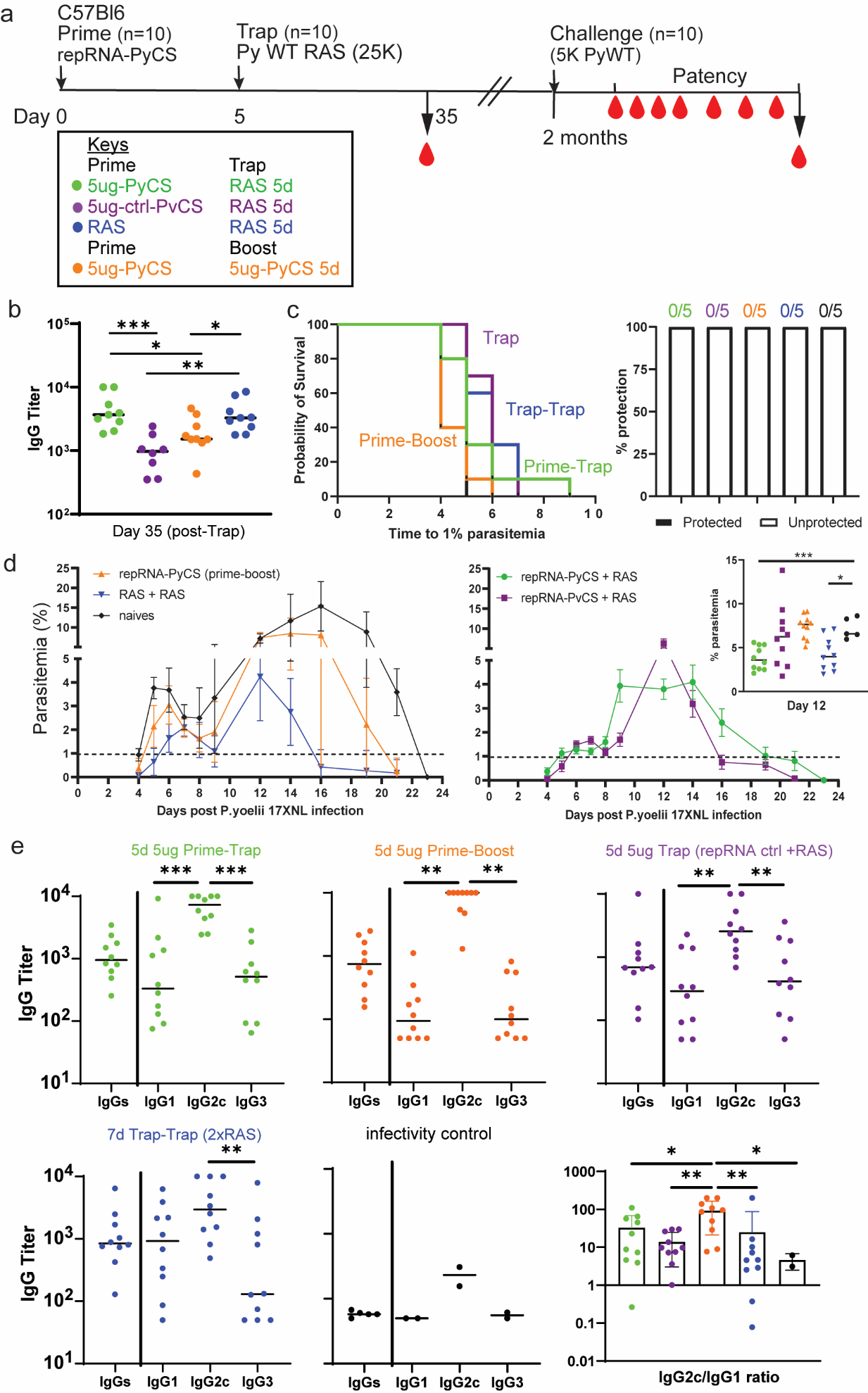
**

**Supplemental Figure 3. Prime-and-trap vaccine in C57BL/6 mice**. a) Schedule of immunization. Prime-and-trap vaccine composed of a 5ug 5-day regimen of prime with repRNA-PyCS (green data) followed by trap dose of 25,000 RAS. Control cohorts are 5ug 5-day prime-Boost repRNA-PyCS (orange data), trap cohort (repRNA-PvCS +RAS, purple data), double-trap (RAS +RAS, dark blue data), and naïve mice. One-month post-trap (day 35), sera were collected. Two months post-trap, mice were challenged intravenously with 5,000 live spz isolated from infected mosquitos. b) Post-trap sera were harvested (day 35) and CS-specific IgG titers were analyzed by ELISA. c) Patency curves (>1% parasitemia) of mice and protection post-challenge per cohort. Number of mice per cohort indicated above bar graph. d) Parasitemia post-challenge of all cohorts. Emphasized is the parasitemia peak at day 12, where each dot represents a mouse, and the bar is the mean of the cohort. e) Final-bleed sera were collected at endpoint (day 87) to evaluate total IgG titers and IgG1, IgG2a, IgG3 subclasses for each cohort by ELISA. Ratio of IgG2a/IgG1 is indicated in bar graph. The n value represents total number of mice tested per cohort, in one experiment. Each data point represents an individual mouse and the bar represents the group mean. All statistical analyses were performed using a Mann Whitney two-tailed test *p=0.05, **p=0.01, ***p=0.001.
