# Supplementary material for: Accelerated prime-and-trap vaccine regimen in mice using repRNA-based CSP malaria vaccine
Source: NPJ Vaccines. 2024 Jan 10;9:12. doi: 10.1038/s41541-023-00799-4 (PMC10781674; doi:10.1038/s41541-023-00799-4)
Supplement: Supplementary file 2 — REPORTING SUMMARY [file 41541_2023_799_MOESM2_ESM.pdf]

nature portfolio

Corresponding author(s): MARION AVRIL

Last updated by author(s): Nov 13, 2023

Reporting Summary

Nature Portfolio wishes to improve the reproducibility of the work that we publish. This form provides structure for consistency and transparency in reporting. For further information on Nature Portfolio policies, see our Editorial Policies and the Editorial Policy Checklist.

Statistics

For all statistical analyses, confirm that the following items are present in the figure legend, table legend, main text, or Methods section.

n/a	Confirmed	
☐	☒	The exact sample size (*n*) for each experimental group/condition, given as a discrete number and unit of measurement
☐	☒	A statement on whether measurements were taken from distinct samples or whether the same sample was measured repeatedly
☐	☒	The statistical test(s) used AND whether they are one- or two-sided<br>*Only common tests should be described solely by name; describe more complex techniques in the Methods section.*
☒	☐	A description of all covariates tested
☒	☐	A description of any assumptions or corrections, such as tests of normality and adjustment for multiple comparisons
☐	☒	A full description of the statistical parameters including central tendency (e.g. means) or other basic estimates (e.g. regression coefficient) AND variation (e.g. standard deviation) or associated estimates of uncertainty (e.g. confidence intervals)
☐	☒	For null hypothesis testing, the test statistic (e.g. *F*, *t*, *r*) with confidence intervals, effect sizes, degrees of freedom and *P* value noted<br>*Give P values as exact values whenever suitable.*
☒	☐	For Bayesian analysis, information on the choice of priors and Markov chain Monte Carlo settings
☒	☐	For hierarchical and complex designs, identification of the appropriate level for tests and full reporting of outcomes
☒	☐	Estimates of effect sizes (e.g. Cohen's *d*, Pearson's *r*), indicating how they were calculated

Our web collection on statistics for biologists contains articles on many of the points above.

Software and code

Policy information about availability of computer code

Data collection	no software was used.
Data analysis	Data analysis was performed as described in the Methods section using the software FlowJo V.10.7.1 (Tree Star),<br>Prism 9.4.1 (GraphPad)

For manuscripts utilizing custom algorithms or software that are central to the research but not yet described in published literature, software must be made available to editors and reviewers. We strongly encourage code deposition in a community repository (e.g. GitHub). See the Nature Portfolio guidelines for submitting code & software for further information.

Data

Policy information about availability of data

All manuscripts must include a data availability statement. This statement should provide the following information, where applicable:

- Accession codes, unique identifiers, or web links for publicly available datasets
- A description of any restrictions on data availability
- For clinical datasets or third party data, please ensure that the statement adheres to our policy

The authors declare that all data generated or analyzed during this study are included in this study are available within the main and supplemental figures.
The data supporting this study’s findings are available from the corresponding authors upon reasonable request.

Research involving human participants, their data, or biological material

Policy information about studies with human participants or human data. See also policy information about sex, gender (identity/presentation), and sexual orientation and race, ethnicity and racism.

Reporting on sex and gender	*Use the terms sex (biological attribute) and gender (shaped by social and cultural circumstances) carefully in order to avoid confusing both terms. Indicate if findings apply to only one sex or gender; describe whether sex and gender were considered in study design; whether sex and/or gender was determined based on self-reporting or assigned and methods used. Provide in the source data disaggregated sex and gender data, where this information has been collected, and if consent has been obtained for sharing of individual-level data; provide overall numbers in this Reporting Summary. Please state if this information has not been collected. Report sex- and gender-based analyses where performed, justify reasons for lack of sex- and gender-based analysis.*
Reporting on race, ethnicity, or other socially relevant groupings	*Please specify the socially constructed or socially relevant categorization variable(s) used in your manuscript and explain why they were used. Please note that such variables should not be used as proxies for other socially constructed/relevant variables (for example, race or ethnicity should not be used as a proxy for socioeconomic status). Provide clear definitions of the relevant terms used, how they were provided (by the participants/respondents, the researchers, or third parties), and the method(s) used to classify people into the different categories (e.g. self-report, census or administrative data, social media data, etc.) Please provide details about how you controlled for confounding variables in your analyses.*
Population characteristics	*Describe the covariate-relevant population characteristics of the human research participants (e.g. age, genotypic information, past and current diagnosis and treatment categories). If you filled out the behavioural & social sciences study design questions and have nothing to add here, write "See above."*
Recruitment	*Describe how participants were recruited. Outline any potential self-selection bias or other biases that may be present and how these are likely to impact results.*
Ethics oversight	*Identify the organization(s) that approved the study protocol.*

Note that full information on the approval of the study protocol must also be provided in the manuscript.

Field-specific reporting

Please select the one below that is the best fit for your research. If you are not sure, read the appropriate sections before making your selection.

☒ Life sciences ☐ Behavioural & social sciences ☐ Ecological, evolutionary & environmental sciences

For a reference copy of the document with all sections, see nature.com/documents/nr-reporting-summary-flat.pdf

Life sciences study design

All studies must disclose on these points even when the disclosure is negative.

Sample size	Sample sizes were sufficient to obtain statistically significant differences between different groups. For challenge experiments, sample size were chosen according to best practices in the field.
Data exclusions	No data were excluded from analysis.
Replication	All experiments were reproduced and the different replicates are clearly labeled in the figures and described in the legends.
Randomization	Mice were randomly allocated from same batch to the experimental groups (mouse age: 6-8 weeks, but always same age within one experiment (+/- 1 day) day)
Blinding	Blinding was not relevant for our study. Measures were taken from sample size with objective readout methods (e.g. ELISA, Flow cytometry, ELIspot) giving numbers as results, which can not manipulated by the person collecting the data.

Reporting for specific materials, systems and methods

We require information from authors about some types of materials, experimental systems and methods used in many studies. Here, indicate whether each material, system or method listed is relevant to your study. If you are not sure if a list item applies to your research, read the appropriate section before selecting a response.

Materials & experimental systems

n/a	Involved in the study	
☐	☒	Antibodies
☐	☒	Eukaryotic cell lines
☒	☐	Palaeontology and archaeology
☐	☒	Animals and other organisms
☒	☐	Clinical data
☒	☐	Dual use research of concern
☒	☐	Plants

Methods

n/a	Involved in the study	
☒	☐	ChIP-seq
☐	☒	Flow cytometry
☒	☐	MRI-based neuroimaging

Antibodies

Antibodies used	Anti-CSP rabbit polyclonal antibodies (antisera) were raised by GenScript (GenScript, NJ). The following Abs were used tfor flow cytometry: CD3e-BUV395 (clone 145-2C11; BD Biosciences), B220-BV711 (clone RA3-6B2; Bio-Legend), CD4–Alexa Fluor 700 (clone GK1.5; BioLegend), CD8a-BV421 (clone 53-607; BD Biosciences), CD69-BV510 (clone H1.2F3; BD Biosciences), CD44–Alexa Fluor 488 (clone IM7; BioLegend), CD62LPE-Cy7 (clone MEL-14; BD Biosciences), KLRG1-PerCP-Cy5.5 (clone 2F1/KLRG1; BioLegend), CXCR6-PE (clone 221002; R&D Systems), and CSP tetramer (CSP epitope SYVPSAEQI provided by the National Institutes of Health Tetramer Core) conjugated to streptavidin-allophycocyanin (ProZyme).
Validation	Antibodies were validated and titrated for specificity prior each study.

Eukaryotic cell lines

Policy information about cell lines and Sex and Gender in Research

Cell line source(s)	Wild-type Py (17XNL strain) parasites (sporozoites) were prepared by cyclical transmission in BALB/cJ mice and Anopheles stephensi mosquitoes at the Seattle Children's Center for Global Infectious Disease Research Insectary (Seattle, WA, USA)
Authentication	To qualify the vaccine candidate in vitro, BHK cells [American Type Culture Collection (ATCC)] were transfected with repRNA or mock transfected.
Mycoplasma contamination	Cell lines were purchased with the certificate of analysis.<br>Cells lines came mycoplasma free from the manufacturer , were directly used and not further tested.
Commonly misidentified lines<br>(See ICLAC register)	*Name any commonly misidentified cell lines used in the study and provide a rationale for their use.*

Animals and other research organisms

Policy information about studies involving animals; ARRIVE guidelines recommended for reporting animal research, and Sex and Gender in Research

Laboratory animals	Mus musculus Balb/cJ female mice (6-8 weeks old) from The Jackson Laboratories (Bar Harbor, ME, USA).
Wild animals	Study did not involve wild animals.
Reporting on sex	No sex based analysis was performed in our study. In the studies ,Balb/cJ only female mice were used .
Field-collected samples	The study did not involve samples collected from the field
Ethics oversight	Mice were maintained under pathogen-free conditions in animal facilities and were fed autoclaved food ad libitum. Mice were housed and cared for in standard IACUC approved animal facilities at Bloodworks Northwest and used in compliance with IACUC approved protocol 5285-01, which adheres to the NIH Office of Laboratory Animal Welfare standards.

Note that full information on the approval of the study protocol must also be provided in the manuscript.

Plants

Seed stocks

Report on the source of all seed stocks or other plant material used. If applicable, state the seed stock centre and catalogue number. If plant specimens were collected from the field, describe the collection location, date and sampling procedures.

Novel plant genotypes

Describe the methods by which all novel plant genotypes were produced. This includes those generated by transgenic approaches, gene editing, chemical/radiation-based mutagenesis and hybridization. For transgenic lines, describe the transformation method, the number of independent lines analyzed and the generation upon which experiments were performed. For gene-edited lines, describe the editor used, the endogenous sequence targeted for editing, the targeting guide RNA sequence (if applicable) and how the editor was applied.

Authentication

Describe any authentication procedures for each seed stock used or novel genotype generated. Describe any experiments used to assess the effect of a mutation and, where applicable, how potential secondary effects (e.g. second site T-DNA insertions, mosiacism, off-target gene editing) were examined.

Flow Cytometry

Plots

Confirm that:

☒ The axis labels state the marker and fluorochrome used (e.g. CD4-FITC).

☒ The axis scales are clearly visible. Include numbers along axes only for bottom left plot of group (a 'group' is an analysis of identical markers).

☒ All plots are contour plots with outliers or pseudocolor plots.

☒ A numerical value for number of cells or percentage (with statistics) is provided.

Methodology

Sample preparation

Mice livers were perfused, mashed into a single cell suspension, and intrahepatic lymphocytes were isolated. Final liver lymphocyte pellets were resuspended in 150 mL 1X MACs buffer (PBS 1 mM EDTA, 0.5% FBS) and transferred to a Ubottom 96-well plate for blocking and staining for flow cytometry.

Instrument

Flow cytometry was conducted on the LSRII instrument (BD Biosciences).

Software

Data were collected using the BD FACSDivaTM software. Cell events were analyzed using FlowJo version 10.7.1 (BD Biosciences).

Cell population abundance

Mice liver lymphocytes were treated with an Fc block and live/dead dye for 30 minutes (anti-CD16/32, clone 2.4G2; BD Biosciences), stained with antibody cocktail for 45 minutes, and fixed for 20 minutes (Cytofix/Cytoperm reagent; BD Biosciences, Franklin Lakes, NJ)

Gating strategy

Gating strategy as demonstrated and published in Watson et al, ASTMH 2022, 106(4). Cells were gated for CD8+ T cells (CD3e+, B220-, CD4-), CD44hi by CD62Llo, then assessed by either KLRG1lo by CD69hi or by CXCR6hi by CD69hi. Antigen specificity was then assessed by PyCSP-tetramer (SYVPSAEQI-specific H2-Kd tetrame). Cell count per gram of tissue was calculated based on a known concentration of counting beads per samples to normalize data.

☐ Tick this box to confirm that a figure exemplifying the gating strategy is provided in the Supplementary Information.
